# A period of 10 weeks of increased protein consumption does not alter faecal microbiota or volatile metabolites in healthy older men: a randomised controlled trial

**DOI:** 10.1017/jns.2020.15

**Published:** 2020-07-03

**Authors:** S. M. Mitchell, E. J. McKenzie, C. J. Mitchell, A. M. Milan, N. Zeng, R. F. D'Souza, F. Ramzan, P. Sharma, E. Rettedal, S. O. Knowles, N. C. Roy, A. Sjödin, K.-H. Wagner, J. M. O'Sullivan, D. Cameron-Smith

**Affiliations:** 1 Liggins Institute, University of Auckland, Auckland, New Zealand; 2 Riddet Institute, Massey University, Palmerston North, New Zealand; 3 Department of Physiology, University of Auckland, Auckland, New Zealand; 4 School of Kinesiology, University of British Columbia, Vancouver, Canada; 5 Food Nutrition & Health Team, AgResearch, Palmerston North, New Zealand; 6 The High-Value Nutrition National Science Challenge, Auckland, New Zealand; 7 Discipline of Nutrition, School of Medical Sciences, University of Auckland, Auckland, New Zealand; 8 Maurice Wilkins Centre for Molecular Biodiscovery, University of Auckland, Auckland, New Zealand; 9 Department of Nutrition, Exercise and Sports, Copenhagen University, Copenhagen, Denmark; 10 Department of Nutritional Sciences and Research Platform Active Ageing, University of Vienna, Vienna, Austria; 11 Singapore Institute for Clinical Sciences, Agency for Science, Technology, and Research, Singapore

**Keywords:** Microbiome, Recommended dietary intake, Volatile organic compounds, Fermentation, 16S amplicon sequencing, 16S rRNA, 16S ribosomal RNA, 2RDA, diet containing twice the RDA of protein, AMDIS, Automated Mass Spectral Deconvolution and Identification System, BCFA, branched-chain fatty acid, HS/SPME/GCMS, head space/solid-phase microextraction/GC-MS, MaAsLin, Multivariate Association with Linear Model, RDA, diet containing the RDA of protein, VOC, volatile organic compound

## Abstract

Diet has a major influence on the composition and metabolic output of the gut microbiome. Higher-protein diets are often recommended for older consumers; however, the effect of high-protein diets on the gut microbiota and faecal volatile organic compounds (VOC) of elderly participants is unknown. The purpose of the study was to establish if the faecal microbiota composition and VOC in older men are different after a diet containing the recommended dietary intake (RDA) of protein compared with a diet containing twice the RDA (2RDA). Healthy males (74⋅2 (sd 3⋅6) years; *n* 28) were randomised to consume the RDA of protein (0⋅8 g protein/kg body weight per d) or 2RDA, for 10 weeks. Dietary protein was provided via whole foods rather than supplementation or fortification. The diets were matched for dietary fibre from fruit and vegetables. Faecal samples were collected pre- and post-intervention for microbiota profiling by 16S ribosomal RNA amplicon sequencing and VOC analysis by head space/solid-phase microextraction/GC-MS. After correcting for multiple comparisons, no significant differences in the abundance of faecal microbiota or VOC associated with protein fermentation were evident between the RDA and 2RDA diets. Therefore, in the present study, a twofold difference in dietary protein intake did not alter gut microbiota or VOC indicative of altered protein fermentation.

Life expectancy is increasing and, with it, a steady rise in the proportion of older adults worldwide^(1)^. Accordingly, it is crucial to understand optimal nutrient intakes, such that wider evidence-based dietary recommendations can be made for the elderly. The current RDA of protein for all adults aged over 19 years according to the WHO^(2)^ and the United States Department of Agriculture^(3)^ is 0⋅8 g protein/kg body weight per d. New evidence suggests that the protein requirements of the elderly should be reconsidered^(4)^, in part to compensate for age-related losses in muscle mass and function (sarcopenia)^(5)^. Ageing alters gastrointestinal tract physiology and function, potentially limiting the digestion of protein^(6)^. Regardless, greater protein intake leads to greater quantities of dietary protein entering the distal gut^(7)^, and this has been linked to the pathogenesis of various chronic disease^(8–11)^.

The gut microbiome is a complex community of micro-organisms, essential for the maintenance of host health^(12)^. Microbial fermentation of nutrients in the lower gut produces a vast range of metabolites that have an impact on the colonic environment, the epithelium, and can be transported to other host tissues^(13)^. Both metabolite production and microbiota composition are highly contingent on nutrient availability^(14,15)^. Although the gut microbiome can also be resilient against short-term dietary change^(16)^, numerous studies have demonstrated some plasticity of the gut microbiome through dietary interventions^(17–19)^.

Carbohydrates are the most studied dietary components relating to gut microbiota composition and metabolite production. Diets rich in non-digestible carbohydrates such as NSP and resistant starch increase the abundance of SCFA, particularly butyrate, and abundance of saccharolytic bacteria (for example, *Bifidobacterium* and *Lactobacillus*). SCFA are an important source of nutrients and protect against cancer and inflammation^(20)^. By contrast, few studies have investigated the effect of high-protein diets on the gut microbiome. Of the current literature, increased dietary protein is only examined along with simultaneous energy deficits and/or coupled with an altered relative proportion of fibre intake, thereby introducing potential significant confounding. In those studies, products of protein fermentation such as faecal branched-chain fatty acids (BCFA), amines, phenols and indoles were increased^(21)^. When produced in high concentrations, metabolites derived from protein fermentation are implicated in DNA damage, inflammation, chronic kidney disease, CVD and cancer^(21,22)^. In addition, high-protein diets reduced SCFA-producing bacteria with a concomitant decline in SCFA^(17,18,23)^. It is likely that the reduction in carbohydrate rather than the increased protein is the culprit here. Indeed, studies have demonstrated the moderating effect of non-digestible carbohydrates on the proteolytic activity of gut microbiota, by increasing SCFA abundance and decreasing proteolytic metabolites such as phenol^(24–27)^. However, evidence for the effects of fibre-rich high-protein diets on gut microbiota and metabolite production is scarce.

Numerous studies have investigated the gut microbiome of elderly populations^(28)^. Profiles of their gut microbiota relative to younger counterparts are characterised by reduced diversity, reduction in beneficial species, and a shift in dominant species^(29)^. However, given the heterogeneity of the elderly population due to lifestyle, living situation and co-morbidities including medication use, a typical microbiota composition is difficult to define^(28)^. Compared with younger adults, concentrations of faecal SCFA tend to be lower in elderly individuals, and studies in elders treated with antibiotics and centenarians have demonstrated a decline in saccharolytic gut bacteria such as *Bifidobacterium* and increased numbers of facultative proteolytic bacteria^(30–32)^. To date, no randomised controlled trials have investigated the impact of increased dietary protein intake on the composition and diversity of the gut microbiome and metabolite production, in the context of an otherwise healthy and balanced diet in the community-dwelling elderly.

In light of the mounting recommendations for increasing the RDA of protein for elderly^(33)^, and the potential for greater proteolytic gut microbial metabolism in this age group, consideration for the implications of increased protein intake on gut health is relevant. Therefore we investigated the impact of a controlled 10-week diet containing either the RDA (0⋅8 g/kg body weight per d) or 2RDA (twice the RDA) of protein in the context of a whole-food diet providing similar quantities per group of fibre, derived from fruits and vegetables, on the composition and diversity of the gut microbiota and production of gut bacteria-derived metabolites. We hypothesised that the 2RDA diet would lead to an increase in protein-fermenting gut bacteria and metabolites of protein fermentation.

## Methods

### Study design

The study was a 10-week randomised parallel-group design. Allocation (1:1 ratio) was conducted by using a locked spreadsheet that assigned participants to treatment groups. The study was not blinded as the investigators were involved in diet preparation, and due to the types of food provided the participants were aware of which group they were assigned. Ethical approval was obtained from the Southern Health and Disability Ethics Committee (New Zealand; 15/STH/236). The trial was conducted according to the Declaration of Helsinki. The study was prospectively registered with the Australian and New Zealand Clinical Trial Registry (www.anzctr.org.au) as ACTRN no. 12616000310460. Informed written consent was obtained from all participants before they were enrolled in the trial. This article reports on secondary outcomes of the OptiMuM (Optimal nutrition in the elderly: High protein diets for muscular, metabolic, and microbiome health) study. The primary outcome for OptiMuM was to evaluate the ability of a high-protein diet to attenuate loss of muscle function and size in healthy ageing males. The secondary outcomes reported in this paper included assessment of faecal microbiota composition and diversity, and faecal metabolites (volatile organic compounds; VOC).

### Participants

A total of thirty-one participants were recruited and thirty were enrolled in the study (Table 1). Eligible participants were males aged 70 years and over, non-smokers, and with a BMI between 20 and 35 kg/m^2^. Participants who enrolled were not taking probiotics for at least 1 month or antibiotics for at least 3 months preceding study commencement. Individuals were excluded from participation if they had a prior history of cancers, diabetes, thyroid disease or gastrointestinal disease. Those with restricted eating habits including vegetarians and those with food allergies or intolerances were also not included in the study. Participants were recruited from Auckland, New Zealand and data were collected at the Liggins Institute, University of Auckland, between March and October 2016. Random assignment of individual participants into each group was performed using sequences generated by www.random.org^(^^34^^)^.

**Table 1.** Participant characteristics pre-intervention (Mean values, standard deviations and ranges; numbers of participants)

Parameter	RDA group				2RDA group			
	Mean	sd	Range	*n*	Mean	sd	Range	*n*
Number of participants*				15				13
Age (years)	74⋅7	3⋅9	70–81		73⋅7	3⋅3	70–79	
Height (cm)	172⋅8	8⋅2	157–187		171⋅7	5⋅5	163–182	
Weight (kg)	85⋅7	20⋅5	49⋅3–111⋅8		83⋅0	8⋅3	70⋅5–98⋅1	
BMI (kg/m^2^)	28⋅4	5⋅1	18⋅9–35⋅3		28⋅2	3⋅3	24⋅1–33⋅9	
Medication usage								
Statin				4				6
ACE inhibitor				4				3
Aspirin				4				2
Ca channel blocker				1				2
PPI				0				2
α-Blocker				1				2
β-Blocker				1				2
Xanthine oxidase inhibitor				1				1

RDA, diet containing the RDA of protein; 2RDA, diet containing twice the RDA of protein; ACE, angiotensin converting enzyme; PPI, proton pump inhibitors.

* Only the participants who completed the study were included in the analysis (*n* 28).

### Study procedures

A detailed description of the study procedures and dietary control has been published previously^(35)^. Briefly, participants were randomised to receive either the RDA (0⋅8 g protein/kg body weight per d) or 2RDA (1⋅6 g protein/kg body weight per d) for 10 weeks. The quantity of protein in the 2RDA diet was chosen as it has previously been recommended as optimal for preserving muscle mass in older adults^(35)^. Both diets provided approximately 30 % of total energy from fat, with the remainder made up from carbohydrate. Protein was provided from a combination of animal and plant sources including dairy products, eggs, poultry, fish, red meat, legumes, grains, nuts and seeds. Both diets contained a similar amount of plant protein whereas the 2RDA diet contained a larger proportion of animal-sourced protein. The two diets were allocated the New Zealand recommendations for daily fruit and vegetable intake (at least five serves per d)^(36)^. The energy content of the diets was calculated to match estimated energy needs for weight maintenance based on the Harris–Benedict equation and adjusted for physical activity level^(37)^ assessed by wrist-worn accelerometers (Fitbit Charge, HR; Fitbit, Inc.). All meals and snacks were provided by the investigators for the duration of the study and delivered weekly to the participants' homes. During the intervention, participants attended the University of Auckland Nutrition and Mobility Clinic at the Liggins Institute at weeks 4, 6 and 8 for weight measurement and compliance check. Participants were asked to make no changes to their exercise routine throughout their participation in the trial.

### Dietary analysis

Individual habitual dietary intake (based on 3-d food diaries) and diets consumed during the trial were analysed using Foodworks software (version 8; Xyris Software Pty Ltd). Compliance was monitored via fortnightly visits and adjusted according to individual preferences within macronutrient allowances to assist with compliance.

### Sample collection

For the analysis of gut microbiota composition and VOC abundance, faecal samples were collected pre-intervention (pre) and after 10 weeks of intervention (post). Participants were provided with a stool collection kit for home collection. Each kit contained a collection vessel to capture the sample, and two collection containers: one containing 2 ml RNA-stabilising solution (RNAlater; Ambion) for bacterial nucleic acid extraction, and another for VOC analyses. Samples were delivered on ice within 2 h of collection via courier to the laboratory at the Liggins Institute. DNA extraction was processed within 5 h of arrival at the laboratory. Faecal samples for the VOC analysis were immediately stored at −80°C for later processing. Uniform sample collection, handling and storage procedures were conducted according to recommendations to minimise bias and differences in detection of microbiota and VOC^(38–40)^.

### Faecal DNA extraction

DNA extraction, bacterial 16S ribosomal RNA (16S rRNA) gene amplification and sequencing of the 16S rRNA gene libraries were performed at the Liggins Institute laboratory. DNA was extracted from faecal samples using the AllPrep DNA/RNA Mini Kit (Qiagen) according to the modified procedures reported by Giannoukos *et al*.^(41)^. The purity of extracted DNA was determined with a NanoDrop^™^ ND-1000 spectrophotometer (NanoDrop Technologies). Extracted DNA was stored (100 μl aliquots; −80°C).

### Microbiota composition analysis by 16S ribosomal RNA amplicon sequencing

DNA concentration was quantified on the Qubit® 2.0 fluorometer (Thermo Fisher Scientific) using the Qubit® dsDNA High Sensitivity Assay Kit (ThermoFisher Scientific). The Ion 16^™^ Metagenomics Kit (Thermo Fisher Scientific) was used to amplify the 16S rRNA gene. The kit contained two different sets of primers targeted to seven hypervariable regions of 16S rRNA: primer set 1 amplified three regions (V2, V4 and V8) and primer set 2 amplified four regions (V3, V6–V7 and V9). Amplicons were fragmented, nick repaired, adaptor ligated, and barcoded for library construction using the Ion Plus Fragment Library Kit (Thermo Fisher Scientific). Libraries were purified using Agencourt AMPure XP magnetic beads (Beckman Coulter). Adaptor-ligated libraries were prepared using the Ion Plus Fragment Library and Ion Xpress^™^ barcode adaptors 1–16 (Thermo Fisher Scientific). Quantification was performed using the Agilent 2100 Bioanalyser with the DNA High Sensitivity Kit (Agilent Technologies). Equimolar concentrations from eight sample libraries (26 pmol/μl) were pooled for each sequencing run. Sequencing was performed on the Ion Torrent Personal Genome Machine (PGM) using the Ion PGM Hi-Q View Sequencing 400 Kit and the Ion 318v2 chip (Thermo Fisher Scientific) at the Liggins Institute laboratory.

### Bioinformatic processing of 16S ribosomal RNA sequence reads

16S amplicon sequences were classified according to the barcode and analysed using the Metagenomics 16S w1.1 workflow in the Ion Reporter software on the Thermo Fisher cloud (version 5.2; Thermo Fisher Scientific) with default parameters^(42)^. After sequencing, the binary alignment map (BAM) files are analysed by Ion Reporter software. Ion Reporter software uses Java scripts to filter the reads by primer and length, before measuring the abundance of each read. Following this, a two-step Basic Local Alignment Search Tool (BLAST) alignment of sequencing reads is performed against both the MicroSEQ (version 2013.1; Thermo Fisher Scientific) and Greengenes (version 13.5) databases. The completed analysis reported the results by consensus for three taxonomic levels (i.e. species, genus and family).

### Volatile organic compound analysis

To determine VOC abundance, triplicates of approximately 200 (range 163–282) mg of frozen faecal material were subsampled from faecal samples that were not exposed to RNAlater, and weighed into tared 20 ml head space vials (Sigma Aldrich). Untargeted extraction of VOC was performed using head space/solid-phase microextraction/GC-MS (HS/SPME/GCMS). Vials were incubated (37°C; 25 min), and volatiles were extracted (37°C; 15 min). The SPME fibres used were divinylbenzene–carboxen–polydimethysiloxane (DVB/CAR/PDMS) 50/30 μm (Supelco), selected for their ability to sample a wide range of VOC. The SPME fibres were pre-conditioned in accordance with the manufacturer's instructions. GCMS analysis was performed using an Agilent GC 7890A with a 5975C inert mass-selective detector (MSD) (Agilent Technologies). The carrier gas was instrument-grade He (99⋅99 %; BOC). The fibre was desorbed in the GC injector in splitless mode, using a butyl rubber septum and a low-volume SPME-specific glass liner (0⋅75 mm internal diameter (ID)) at 250°C for 1 min. A general-purpose column was chosen for compound separation: an Rtx-5Sil MS 30 m, 0⋅25 mm ID, with a 0⋅25 μm stationary phase (95 % dimethylpolysiloxane, 5 % diphenyl; Shimadzu). Column flow was set (1 ml/min), with a column head pressure (7⋅2 psi (pound per square inch)), to provide an average linear velocity of 36 cm/s. The SPME fibre remained in the injector for 5 min to condition for the next run. Purge flow (50 ml/min) commenced 1 min after injection. The GC oven was initially set at 35°C for 5 min, increasing to 100°C at 5°C/min, further to 200°C at 15°C/min, and finally to 300°C at 30°C/min, before being held for 3 min, with a total run time of 31 min. The detector source was maintained at 230°C and the quadrupole at 150°C. The detector was run in positive-ion, electron-impact ionisation mode, at 70 eV. Data were acquired at 1789 atomic mass units (amu)/s in scan mode, with a mass range of 24 to 300 amu, and zero threshold.

### Volatile organic compound data processing

Deconvolution and identification of compounds were performed using the Automated Mass Spectral Deconvolution and Identification System (AMDIS, version 2.71)^(43)^. The AMDIS limitation on mass spectral library size was circumvented by developing a smaller subset library from the National Institute of Standards and Technology (NIST) main mass spectral library^(44)^. The subset library was constructed using Agilent MSD Productivity ChemStation (version F.01.01.2317) setting integration parameters to be sensitive to low-abundance compounds, and search-match parameters to be expansive. The top five identities for each peak, for all peaks, and for all samples were combined to construct a subset library of 17 901 mass spectra, which were then used with AMDIS. The settings for AMDIS were optimised to maximise annotation of all features, including unknowns, reducing the false-negative rate to <5 %. Mass spectral matching was used to assign identities. Matches <60 % are considered unknown and were excluded from analysis.

Matches between 80 and 100 % match to a reference standard can be considered putatively identified, those with a 60–79 % mass spectral match can be considered tentatively identified, and those with <60 % mass spectral match can be considered unknown^(45)^. Data were filtered to exclude compounds that were identified at a low frequency (<9 identifications/156 samples) in the dataset. For peak integration an R-script based on XCMS (MassOmics, version 2.5)^(46)^ was used, with the retention time bins defined by AMDIS. The data produced represent a set of probable identifications for each feature, which were then filtered, based on frequency, retention time reproducibility, and library match factor to assign the most probable identification. Co-eluting peaks were resolved and data were checked against negative controls (empty vials) to identify and remove background contaminants.

### Statistical analysis

The sample size (fifteen participants per group) was calculated based on the power required for the primary outcome for the original study, which was to detect a between-group difference of 800 g whole-body lean mass change in skeletal muscle mass and strength^(35)^. Previous studies investigating the effect of high-protein diets on the gut microbiome demonstrated significant changes in gut microbiota taxa and faecal metabolites in similar samples sizes (*n* 14^(47)^, and *n* 17^(17)^).

Changes in dietary intake were assessed by two-way repeated-measures ANOVA with time (pre- compared with post-intervention) as a repeated factor and diet (RDA compared with 2RDA) as a between-subject factor. ANOVA was performed using SPSS version 25.0 (SPSS Inc.).

For microbiota analysis, comparisons of α-diversity were made by Kruskal−Wallis tests using the Shannon and Simpson indices to determine within-sample richness and evenness. To determine whether the level of variability within groups was not greater than between groups, homogeneity of group dispersions (PERMDISP) was performed. β-Diversity was assessed by permutational multivariate ANOVA (PERMANOVA). Principal coordinate analysis (PCoA) was applied to illustrate the variation present between groups. Statistics and data visualisation for microbiota analysis were carried out with the use of MicrobiomeAnalyst^(48)^.

For the VOC analysis, triplicate samples were averaged by the median and raw data were normalised by the auto scaling method (mean-centred and divided by the standard deviation of each variable). Estimation of main or interaction effects was calculated using ANOVA simultaneous component analysis (ASCA). Univariate analyses of post-intervention results were made by the Wilcoxon–Mann–Whitney test. Principal components analysis (PCA) was applied to illustrate variation between groups post-intervention. Statistics for VOC analysis were performed using MetaboAnalyst v4.0^(49)^. Multivariate Association with Linear Models (MaAsLin) was used to identify significant associations between microbiota taxa and VOC abundance^(50)^. MaAsLin is a type of multivariate statistical analysis to find associations between clinical metadata and microbial composition or functional data. Where appropriate, *P* values were corrected for multiple testing using false discovery rate according to the Benjamini–Hochberg correction. Significance was set at *P* ≤ 0⋅05. Mean values and standard deviations are shown in the tables and text.

## Results

**Fig. 1.**
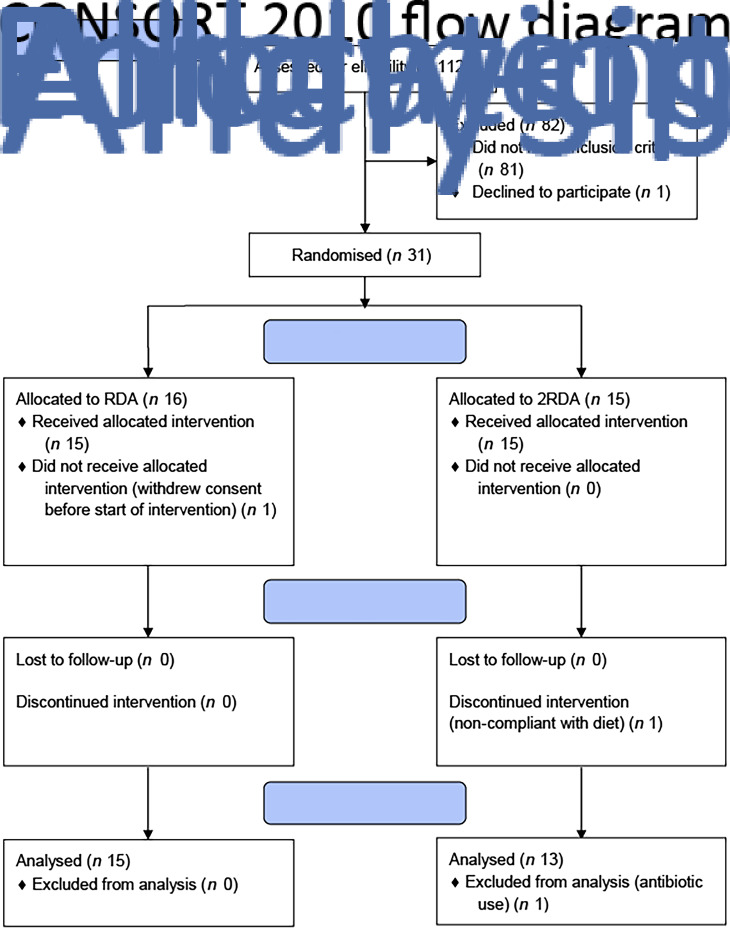
CONSORT (Consolidated Standards of Reporting Trials) diagram. RDA, diet containing the RDA of protein; 2RDA, diet containing twice the RDA of protein.

A total of thirty-one participants were recruited to the study (RDA, *n* 15, 2RDA, *n* 16; Fig. 1). One subject dropped out before commencement of the study (2RDA, *n* 1) and one was excluded due to non-compliance (2RDA, *n* 1). One participant was excluded from the final analysis due to antibiotic use (2RDA, *n* 1). The remaining twenty-eight participants were included in this analysis (RDA, *n* 15; 2RDA, *n* 13). Participant characteristics are reported in Table 1.

### Dietary intake

A detailed account of dietary intake is shown in Table 2. Overall, participants were highly compliant with both diets (compliance for protein intake was 98⋅9 % in the RDA group and 97⋅5 % in the 2RDA group). Compliance was documented in fortnightly food records and according to urinary N excretion assessed pre- and post-intervention (RDA: from 1⋅3 (sd 0⋅2) to 0⋅9 (sd 0⋅2) g/kg body weight per d, *P* = 0⋅001; 2RDA: from 1⋅3 (sd 0⋅2) to 1⋅5 (sd 0⋅1) g/kg body weight per d, *P* = 0⋅001). Average protein intake was altered by the intervention (time × diet interaction *P* < 0⋅001); it reduced in the RDA group (from 104⋅8 (sd 30) to 80⋅1 (sd 23) g/d, *P* = 0⋅004) and increased in the 2RDA group (from 95⋅6 (sd 20) to 136⋅2 (sd 18) g/d; *P* < 0⋅001). Dietary fibre increased (time effect, *P* < 0⋅001) for RDA (from 34 (sd 9⋅9) to 47 (sd 8⋅4) g/d; *P* < 0⋅001), and for 2RDA (from 28⋅9 (sd 8⋅1) to 50⋅3 (sd 5⋅3) g/d; *P* < 0⋅001) but was not different between the diets (*P* = 0⋅410).

**Table 2.** Estimated composition of baseline (pre) and experimental diets (post) for the RDA and 2RDA groups (Mean values and standard deviations)

Nutrient intake	RDA				2RDA				Effect (*P*)§		
	Pre		Post		Pre		Post				
	Mean	sd	Mean	sd	Mean	sd	Mean	sd	Time	Diet	Time × diet
Energy intake									0⋅705	0⋅06	0⋅003*
kJ/d	13 104	4418	11 276‡	2502	9305†	2707	11 627‡	715			
kcal/d	3132	1056	2695‡	598	2224†	647	2779‡	171			
Protein intake											
Total (g/d)	101	30	74‡	15	88	25	136†‡	10	0⋅041*	0⋅001*	<0⋅001*
Relative (g/kg per d)	1⋅2	0⋅4	0⋅9‡	0⋅1	1⋅1	0⋅3	1⋅7†‡	0⋅1	0⋅056	<0⋅001*	<0⋅001*
Animal sourced (g/d)	58	20	35‡	11	53	23	97†‡	6	0⋅016	<0⋅001*	<0⋅001*
Plant sourced (g/d)	43	16	48	9	35	9	47	4	0⋅002*	0⋅177	0⋅185
Estimated by N balance (g/kg per d)	1⋅3	0⋅2	0⋅9‡	0⋅2	1⋅3	0⋅2	1⋅5†‡	0⋅1	0⋅098	<0⋅001*	<0⋅001*
Energy from protein (%)	13⋅7	4⋅4	11⋅2‡	1⋅5	16⋅5	4⋅4	19⋅7†‡	1⋅6	0⋅644	<0⋅001*	0⋅001*
Carbohydrate intake											
Total (g/d)	288	107	368	94	264	102	340	30	0⋅001*	0⋅309	0⋅946
Relative (g/kg/d)	3⋅5	1⋅2	4⋅4	1⋅0	3⋅3	1⋅5	4⋅2	0⋅6	0⋅001*	0⋅493	0⋅915
Energy from carbohydrate (%)	37⋅7	9⋅8	54⋅4‡	2⋅5	47⋅2†	10⋅5	49⋅0†	2⋅2	<0⋅001*	0⋅217	0⋅001*
Fibre intake (g/d)	34	14	57‡	9	33	15	50‡	5	<0⋅001*	0⋅236	0⋅306
Fruit and vegetables (serves/d)	5⋅4	3⋅0	8⋅6‡	2⋅1	4⋅5	2⋅1	8⋅5‡	1⋅6	<0⋅001	0⋅41	0⋅535
Fat intake											
Total (g/d)	161	86	91‡	19	75†	31	84	6	0⋅015*	0⋅002*	0⋅002*
Energy from fat (%)	44⋅6	11⋅0	30⋅4‡	1⋅9	30⋅4†	7⋅3	27⋅2†	1⋅3	<0⋅001*	<0⋅001*	0⋅006*
Saturated fat (g/d)	57	27	33‡	8	29†	13	28†	4	0⋅005*	<0⋅001*	0⋅011*
Cholesterol (mg)	387	142	299	83	351	113	527	73	0⋅106	0⋅003	<0⋅001
Alcohol (g/d)	8⋅8	15⋅7	–		9⋅6	8⋅6	–		–	0⋅236	–

RDA, diet containing the RDA of protein; 2RDA, diet containing twice the RDA of protein.

* Significant main effect or interaction (*P* < 0⋅05).

† Different between diets at indicated time point. *P* values were controlled using the Sidak *post hoc* procedure.

‡ Different from pre-intervention within the same group (*P* < 0⋅05).

§ Main effects and interactions were calculated by two-way repeated-measures ANOVA. Modified from Mitchell *et al*.^(35)^, used with permission.

### Microbial taxa composition

**Fig. 2.**
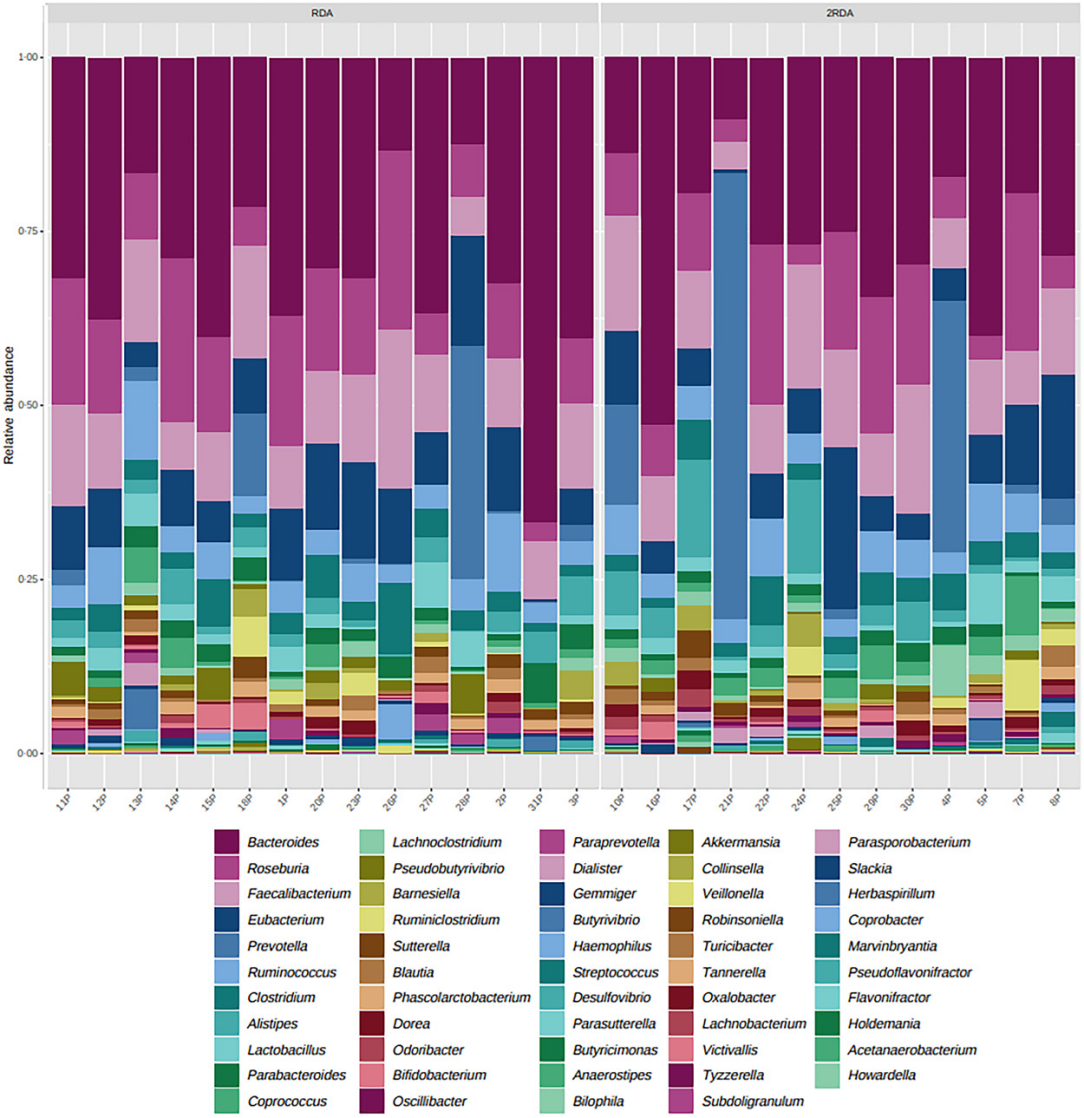
Microbiota composition. Relative abundance at the genus level post-intervention in the RDA and 2RDA groups. RDA, diet containing the RDA of protein; 2RDA, diet containing twice the RDA of protein.

**Fig. 3.**
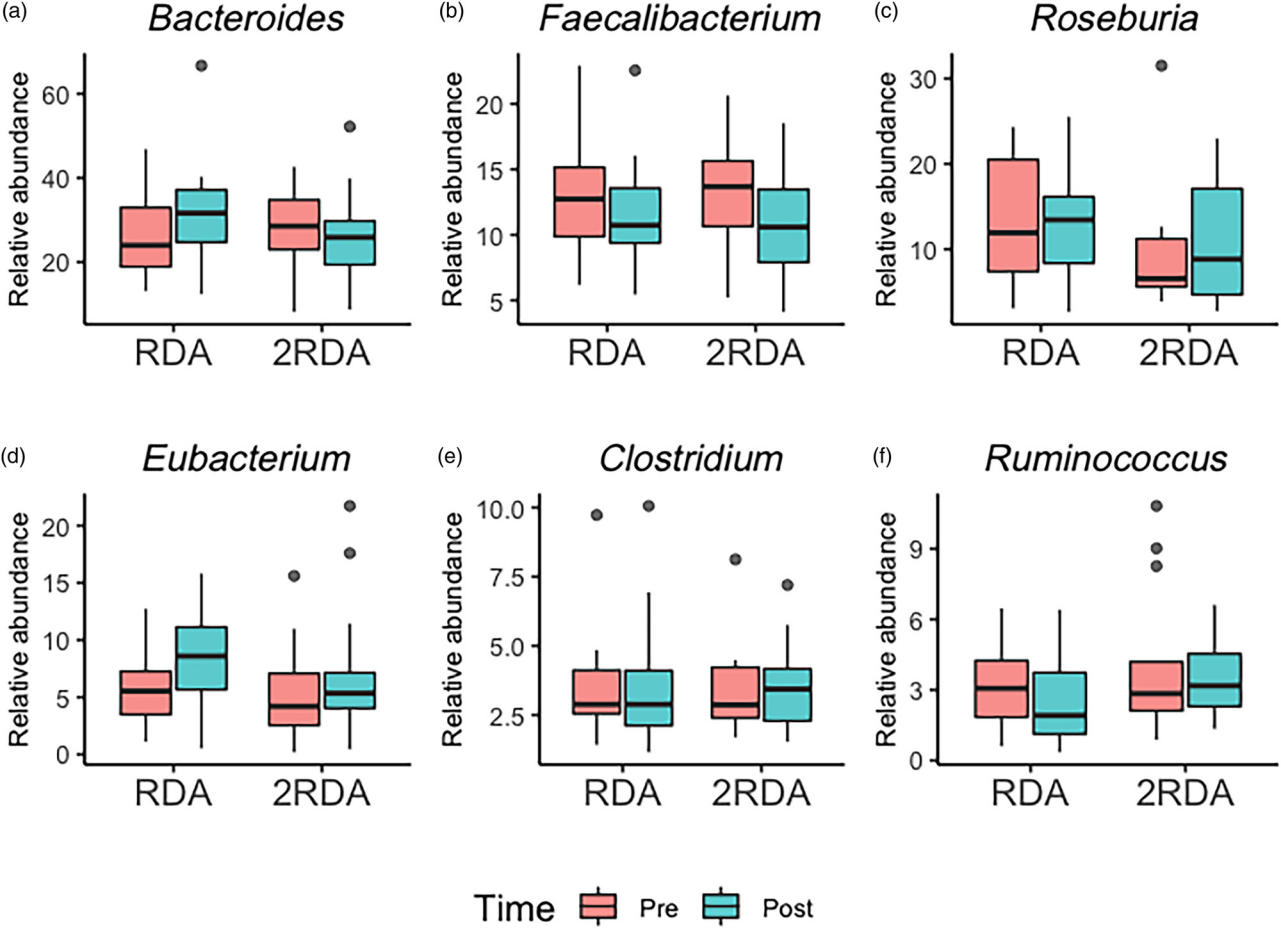
Genera boxplots: the most abundant genera in the RDA and 2RDA groups. RDA, diet containing the RDA of protein; 2RDA, diet containing twice the RDA of protein. For the box plots: middle line is the median, boxes represent 25th and 75th percentiles, whiskers are within 1⋅5 interquartile ranges of the lower and upper percentiles, and dots represent outliers.

An average of 398 372 filtered 16S rRNA sequence reads were obtained per sample. Library size was rarefied to a minimum read depth of 189 549. When corrected for multiple testing, there were no differences in relative abundance observed at the phylum, family, genus or species level between groups (genus level post-intervention illustrated in Fig. 2). The most abundant genera in both groups pre- and post-intervention were *Bacteroides*, *Faecalibacterium*, *Roseburia*, *Eubacterium*, *Clostridium* and *Ruminococcus* (Fig. 3).

### Microbial diversity

**Fig. 4.**
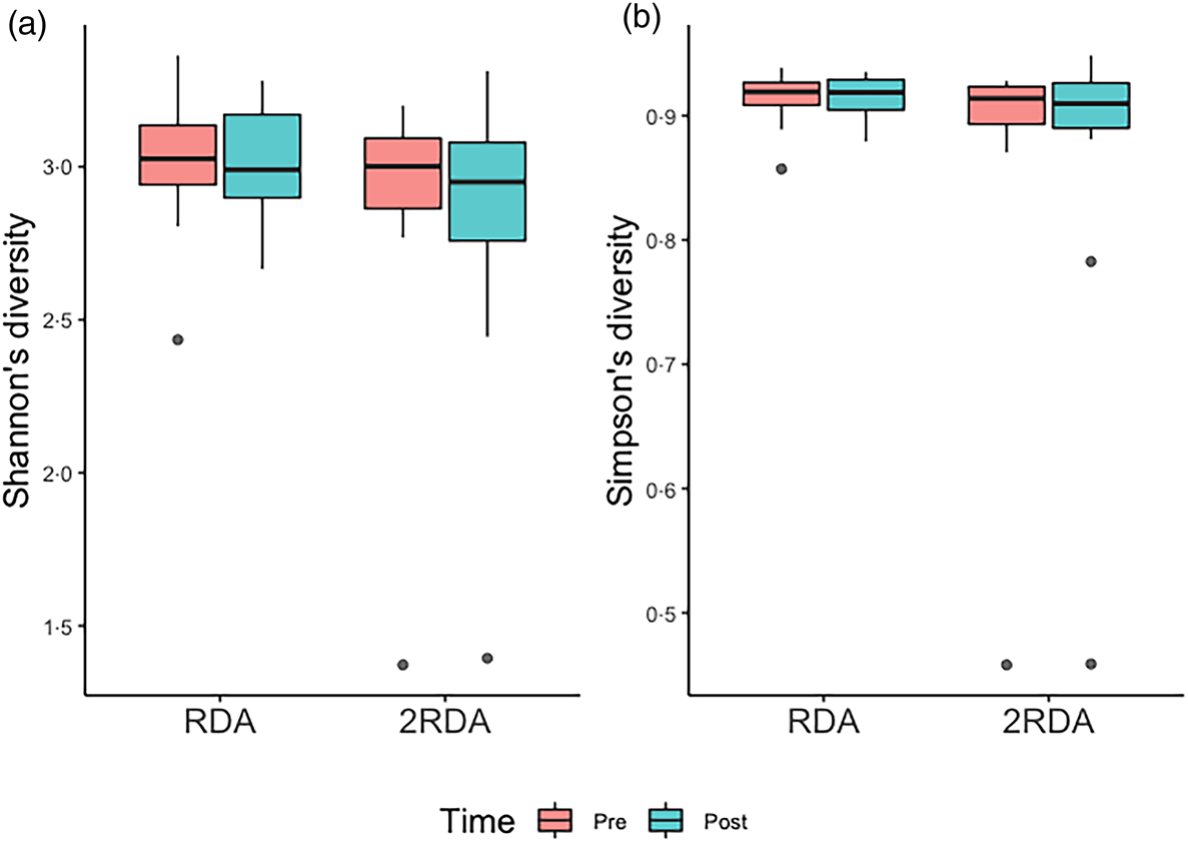
α-Diversity. (a) Shannon's diversity; (b) Simpson's diversity. RDA, diet containing the RDA of protein; 2RDA, diet containing twice the RDA of protein. For the box plots: middle line is the median, boxes represent 25th and 75th percentiles, whiskers are within 1⋅5 interquartile ranges of the lower and upper percentiles, and dots represent outliers.

**Fig. 5.**
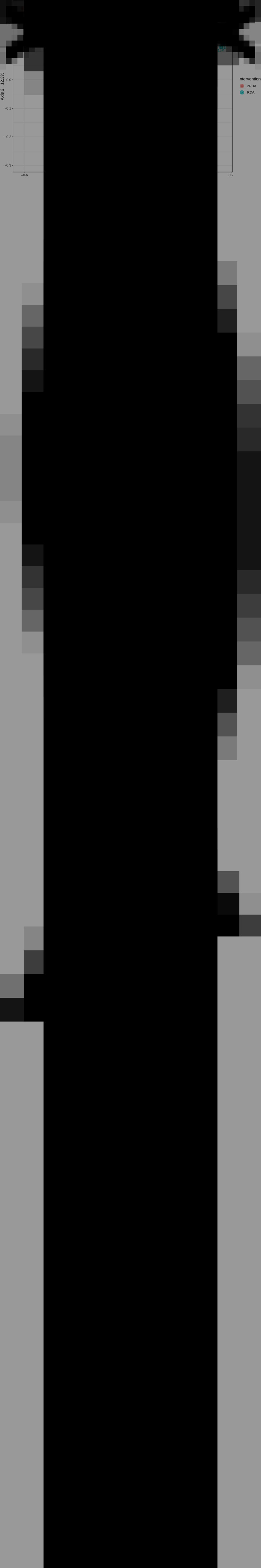
β-Diversity. RDA, diet containing the RDA of protein; 2RDA, diet containing twice the RDA of protein.

α-Diversity was assessed using the Simpson and Shannon indices (Fig. 4). There was no change over time in α-diversity for the RDA (Shannon, *P* = 0⋅632; Simpson, *P* = 0⋅935) or the 2RDA groups (Shannon, *P* = 0⋅250; Simpson, *P* = 0⋅945). There was also no significant difference in α-diversity between groups pre-intervention (Shannon, *P* = 0⋅650; Simpson, *P* = 0⋅339) or post-intervention (Shannon, *P* = 0⋅548; Simpson, *P* = 0⋅525). Between groups, there was no difference in β-diversity (*P* = 0⋅154; Fig. 5), and the graph demonstrates that individuals (pre- and post-samples) clustered more strongly together than the diet groups. PERMDISP revealed no variability within groups (*P* = 0⋅284).

### Faecal volatile organic compound abundance

**Fig. 6.**
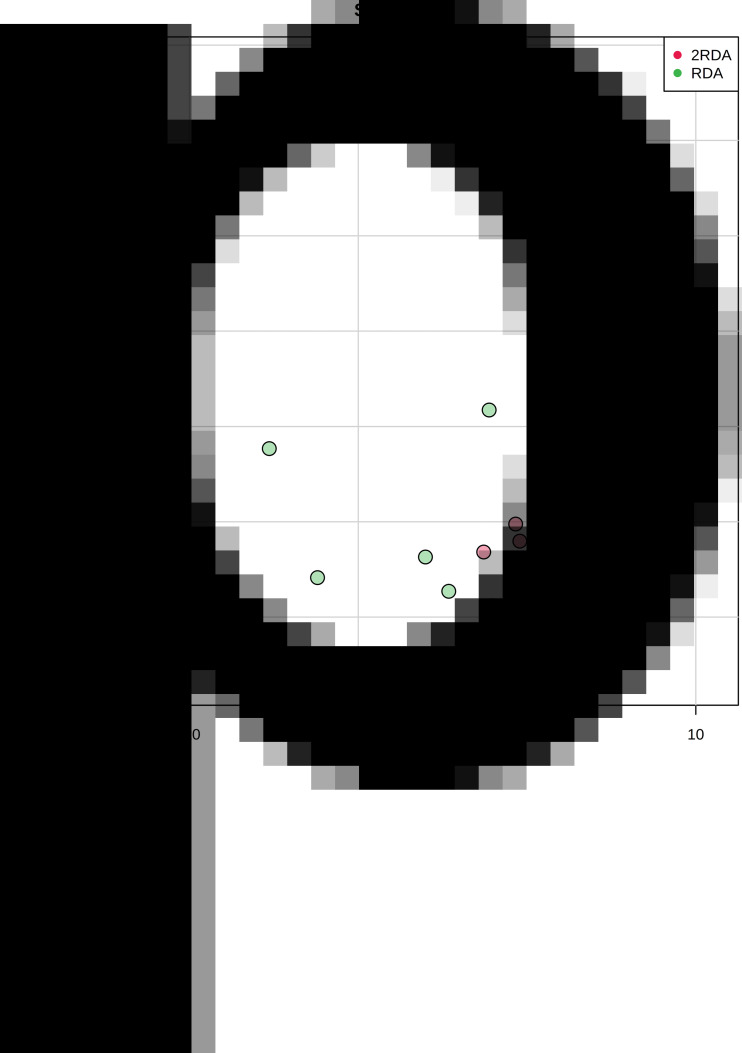
Principal component (PC) analysis of volatile organic compounds. RDA, diet containing the RDA of protein; 2RDA, diet containing twice the RDA of protein.

Untargeted faecal metabolite analysis identified 261 VOC. These were collectively derived from a combination of ingested compounds (for example, from food or drink, inhaled, absorbed) or endogenous compounds (for example, from the microbial fermentation process). A representative list of VOC accompanied by a univariate analysis of post-intervention results is shown in Supplementary Table S1. After adjustment for multiple testing, there were no significant differences in any of the compounds related to bacterial fermentation of dietary components between groups post-intervention. Clustering of VOC for each group post-intervention was plotted by principal components analysis (PCA) (Fig. 6) which showed no differential clustering between groups.

**Fig. 7.**
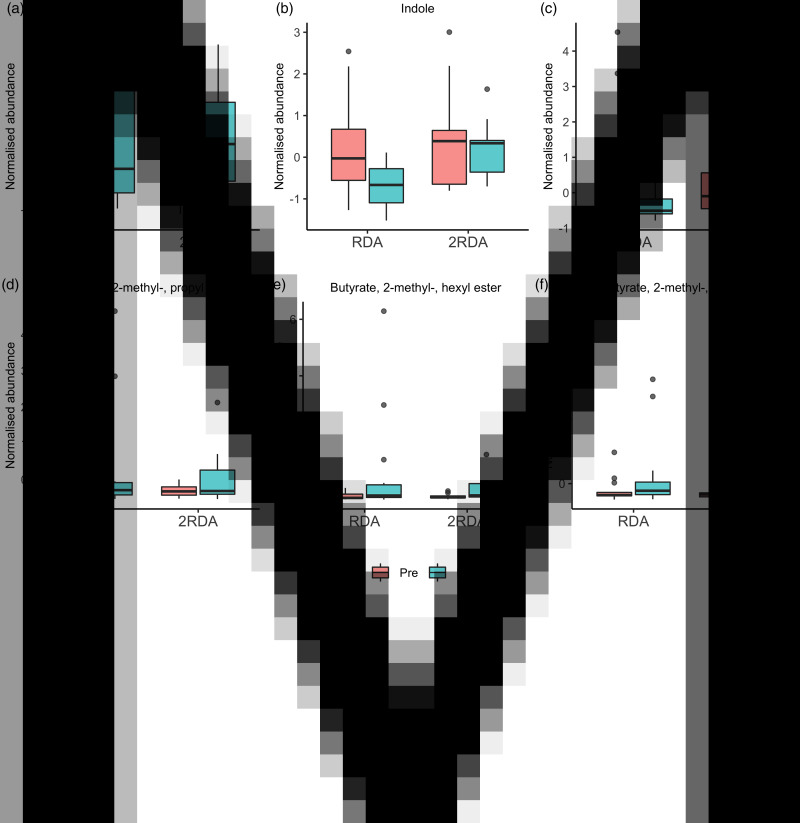
Volatile organic compound boxplots. RDA, diet containing the RDA of protein; 2RDA, diet containing twice the RDA of protein. For the box plots: middle line is the median, boxes represent 25th and 75th percentiles, whiskers are within 1⋅5 interquartile ranges of the lower and upper percentiles, and dots represent outliers.

According to the ANOVA simultaneous component analysis (ASCA), permutation test statistics showed that there were no significant effect for time (*P* = 0⋅497), diet (*P* = 0⋅440) or interaction (time × diet) *P* = 0⋅756). There were six compounds of microbial metabolism well modelled by the main effect of time (Table 3 and Fig. 7). Indole increased in the RDA group from pre- to post-, and relative to the 2RDA group. In both groups, 3-methylbutanal decreased, while the fatty acid esters butyrate, 2-methyl-, hexyl ester; butyrate, 2-methyl-, propyl ester; and butyrate, 2-methyl-, butyl ester all increased in both groups.

**Table 3.** Important features identified by ANOVA simultaneous component analysis (ASCA) related to microbial fermentation*

VOC	Leverage	SPE
Indole	0⋅018	0
3-Methylbutanal	0⋅018	8⋅63 × 10^−32^
Butyrate, 2-methyl-, hexyl ester	0⋅014	8⋅63 × 10^−32^
2-Butanol	0⋅013	8⋅63 × 10^−32^
Butyrate, 2-methyl-, propyl ester	0⋅012	0
Butyrate, 2-methyl-, butyl ester	0⋅011	1⋅08 × 10^−31^

VOC, volatile organic compound; SPE, squared prediction error.

* Table includes the compounds well modelled by the main effect of time.

### Correlations

MaAsLin analysis between 16S and VOC data revealed no significant correlations between microbial taxa (at family, genus or species level) and VOC abundance.

## Discussion

The diet is an important modulator of the gut microbiome and its metabolites^(51)^, with potential important implications for health in an ageing population^(30)^. Here, we collected and analysed faecal samples for microbial DNA and VOC of bacterial fermentation origin, with the aim of characterising the impact of a high-protein diet on healthy older males. The present data indicate that contrary to the hypothesis, a diet containing twice the current RDA of protein, in conjunction with high fibre intake, did not modify the gut microbiota composition nor microbiota-derived VOC production after 10 weeks.

Overall, there was no statistically significant difference in the composition and diversity of the faecal microbiota between groups post-intervention. Ours was the first study to test the effects of a high-protein diet using whole foods as a protein source rather than supplements, while simultaneously increasing fibre intake. The present result is in accord with two short-term dietary studies which investigated a high-protein diet with no change in fibre intake^(52,53)^. Beaumont *et al*.^(52)^ detected no difference in gut microbiota composition of participants consuming for 3 weeks a daily casein or soya supplement (total protein 34 and 31 % of energy and total fibre intake 14⋅4 and 17⋅9 g/d, respectively), compared with a control group (total protein 14 % of energy and total fibre 17 g/d) in a randomised parallel design^(52)^. Similarly, Windey *et al*.^(53)^ observed no effect between a control diet (total protein 15 % of energy intake and total fibre 16⋅3 g/d) and a high-protein diet (total protein 27 % of energy intake and total fibre 15⋅4 g/d) during 2 weeks in a randomised cross-over design^(53)^. In contrast, where protein intake has increased, concurrent with a reduction in dietary fibre, significant increases in *Bacteroides*^(54)^, and reductions in butyrate-producing bacteria including *Roseburia*, *Eubacteria* and *Bifidobacterium*, have been reported^(17,18,23)^. It is a reasonable interpretation that these observed differences were chiefly driven by alterations in the total malabsorbed carbohydrate, including dietary fibre, rather than the protein intake *per se*. These data support dietary fibre as a preferred fermentative substrate for microbiota^(55)^, even in the presence of substantial alterations in total protein intake and therefore potential increased protein malabsorption.

The VOC detected by HS/SPME/GCMS were not different in the 2RDA group relative to the RDA group. Protein fermentation by gut microbiota can result in a variety of compounds depending on the amino acids available. In the RDA group we observed a decrease in indole abundance over time, with no change in the 2RDA group. Indole is produced by a variety of microbiota species particularly from the *Bacteroides* genera^(56)^ by the fermentation of tryptophan^(57)^, an amino acid abundant in dairy products, red meat, eggs, fish and poultry. Overproduction of indole can lead to increased production of indoxyl sulphate, a uraemic toxin associated with chronic kidney disease^(58)^. Indole production is likely to have decreased in the RDA group due to the decreased quantity of tryptophan-rich foods in their diet. Indole did not increase in the 2RDA group possibly due to the preferential fermentation of fibre by gut microbiota, as indole production in particular is inhibited in the presence of fermentable carbohydrate^(59)^. The BCFA 2-methylbutyrate, 3-methylbutyrate and isobutyrate are exclusively derived from microbial fermentation of the branched-chain amino acids (isoleucine, leucine and valine)^(60)^. For this reason BCFA abundance is often used as a biomarker for protein fermentation in the gut^(53)^, and increased BCFA abundance has been observed after high protein intake^(17,52)^. However, we did not observe an elevated abundance of BCFA in the 2RDA group relative to the RDA group, although some intermediary products of branched-chain amino acid catabolism were changed in both groups. 3-Methylbutanal (an oxygenated aldehyde) decreased in both groups over time. 3-Methylbutanal is an intermediate product of leucine degradation from *Bacteroides* taxa in the production of the BCFA 3-methylbutyrate^(61)^. In contrast, the carboxylic ester derivatives of the BCFA 2-methylbutyrate tended to increase in both groups over time. However, these esters are also common flavouring additives in foods^(62)^ and therefore it is uncertain if their abundance is reflective of microbial fermentative activity or dietary intake.

The main SCFA (butyrate, acetate and propionate), indicative measures of dietary fibre intake, were not altered despite the increased fibre intake in both study arms. As there are no other studies that have increased protein and fibre intake concurrently, comparison with previous research is challenging. Previous high-protein diet studies have demonstrated that SCFA, particularly butyrate, tend to decrease after a high-protein diet^(17,18,63)^, but these diets were accompanied by low fibre intake. Even a high-protein diet, without alteration in dietary fibre, decreased faecal butyrate concentrations^(52)^. The present findings are interesting, as it is well documented that increasing dietary fibre results in increased butyrate production in particular^(64)^, although a previous study with rats demonstrated that there is no linear correlation between fibre intake and SCFA production^(65)^. Regardless, due to the main site of production – the proximal colon – detection in faecal samples may not be wholly indicative of SCFA abundance. Additionally, SCFA are readily utilised by enterocytes or taken up via systemic circulation. The detection of increased SCFA in faecal samples is typically due to excessive production^(66)^; however, it is conceivable that metabolism of SCFA in the elderly is altered, possibly due to changes in SCFA-producing bacteria and decreased microbial diversity^(29)^. As we did not include a younger cohort in this study by way of comparison, it is unclear if that is the case.

There was a high degree of inter-individual variation of gut microbiota composition, which persisted through the dietary intervention for both groups and was the predominant source of variation in the data. The degree of inter-individual variation could have been controlled by at least two approaches. First, using a cross-over study design reduces the variation at baseline as each participant serves as their own control. Although, as noted, a previous study employing a cross-over design presented similar findings to ours^(52)^. Second, subtle changes may have gone undetected in the small sample size. Indeed, a retrospective power calculation indicated that in order to detect at least a 30 % effect, a total of ninety participants was required (forty-five per group). Elderly people are a heterogeneous population with large variability in physiological health, medication usage and lifestyle. This work investigated a small healthy cohort, which may not account for variations in gut microbial composition and metabolism in elderly accompanying co-morbidities or sex differences, or the frail and institutionalised.

Impaired capacity for nutrient absorption in the gastrointestinal tract may occur in the elderly; however, the literature is conflicting on whether digestion and absorption of protein are affected in healthy older adults^(67)^. As reported previously from this study, whole-body lean mass in the 2RDA group increased relative to the RDA group^(35)^, suggesting in part that greater absorption accompanies increased intake. Indeed, the primary protein sources that were increased in the 2RDA diet were dairy and animal proteins which are highly digestible^(68)^. Regardless, there will be malabsorption of protein and this will be approximately proportional to the amount of protein consumed^(69,70)^. Therefore, it can be reasonably expected that the metabolites from protein fermentation should be higher in the 2RDA group relative to the RDA group, and present in the faeces. In this study we did not detect evidence for this in the faeces using the HS/SPME/GCMS method, therefore future studies may warrant closer examination of the bacterial N utilisation and/or corresponding measurement of absorbed bacterial metabolites and co-metabolites in the urine and/or plasma^(71)^. Indeed, as reported in a separate paper, we also observed increased concentrations of circulatory trimethylamine *N*-oxide (TMAO) in the 2RDA group^(72)^. TMAO is a co-metabolite, generated in the liver after gut microbial metabolism of choline and l-carnitine, nutrients that tend to be highest in protein-rich foods.

### Conclusion

The present results suggest that consuming a diet with twice the RDA of protein for 10 weeks does not result in significant differences in proteolytic microbiota or metabolites of protein fermentation relative to the RDA of protein while achieving the recommended daily intake for fruits and vegetables. There was considerable inter-individual variation in both microbiota composition and VOC, which persisted through the intervention. Moreover, the artificial way by how every meal was provided is not indicative of real-world situations, although provision of protein and fibre through whole foods remains a unique strength. This study was not powered to detect subtle changes, therefore investigation of the interaction of protein intake on the gut microbiota is required within a larger cohort, and inclusive of the variations in dietary quality evident in a free-living elderly population. Such studies will be necessary to comprehensively appraise how dietary protein has an impact on microbial composition and function in the elderly.

## Supplementary material

For supplementary material accompanying this paper visit http://dx.doi.org/10.1017/jns.2020.15. click here to view supplementary material
