# Supplementary material for: A period of 10 weeks of increased protein consumption does not alter faecal microbiota or volatile metabolites in healthy older men: a randomised controlled trial
Source: J Nutr Sci. 2020 Jul 3;9:e25. doi: 10.1017/jns.2020.15 (PMC7372166; doi:10.1017/jns.2020.15)
Supplement: Supplementary file 1 [file S2048679020000154sup001.docx]

**Supplementary Table S1.** Putatively identified VOCs from HS/SPME/GCMS analysis of the OptiMuM study

|  | VOC | *P*-value | *q-*value (FDR) | 2RDA/RDA | |
| --- | --- | --- | --- | --- | --- |
| **Alcohols** | |  |  | |  |
|  | 1-Butanol | 0.751 | 0.961 | | Down |
|  | 1-Butanol, 2-methyl- | 0.751 | 0.961 | | Down |
|  | 1-Butanol, 3-methyl- | 0.169 | 0.707 | | Up |
|  | 1-Hexanol | 0.13 | 0.705 | | Up |
|  | 1-Octanol | 0.785 | 0.973 | | Down |
|  | 1-Octen-4-ol | 0.716 | 0.961 | | Down |
|  | 1-Pentanol | 0.856 | 0.979 | | Up |
|  | 1-Penten-3-ol | 0.413 | 0.84 | | Up |
|  | 1-Propanol | 0.716 | 0.961 | | Up |
|  | 1-Propanol, 2-methyl- | 0.052 | 0.579 | | Down |
|  | 2-Butanol, R- | 0.927 | 0.986 | | Up |
|  | 2-Decen-1-ol, E- | 0.273 | 0.735 | | Up |
|  | 3-Buten-1-ol, 3-methyl- | 0.097 | 0.7 | | Up |
|  | 3-Pentanol | 0.028 | 0.579 | | Up |
|  | Benzyl alcohol | 0.716 | 0.961 | | Down |
|  | Cyclododecanol | 0.217 | 0.707 | | Up |
|  | Cyclohexanol, 5-methyl-2-1-methylethyl-, 1-alpha,2-beta,5-alpha-/-- | 0.716 | 0.961 | | Down |
|  | E-2-Hexadecacen-1-ol | 0.683 | 0.961 | | Down |
|  | Ethanol | 1 | 1 | | Down |
|  | Ethanol, 2-methylphenylmethylamino- | 0.555 | 0.944 | | Up |
|  | Eucalyptol | 0.785 | 0.973 | | Down |
|  | Methyl Alcohol | 0.891 | 0.986 | | Up |
|  | Phenylethyl Alcohol | 0.294 | 0.738 | | Up |
|  | Terpinen-4-ol | 0.235 | 0.707 | | Down |
|  | trans-2-Undecen-1-ol | 0.097 | 0.7 | | Up |
| **Aldehydes** | |  |  | |  |
|  | Acetaldehyde | 0.555 | 0.944 | | Up |
|  | Benzaldehyde | 0.751 | 0.961 | | Up |
|  | Benzeneacetaldehyde | 0.963 | 0.993 | | Up |
|  | Butanal | 0.021 | 0.579 | | Up |
|  | Butanal, 3-methyl- | 0.65 | 0.961 | | Up |
|  | Dodecanal | 0.439 | 0.882 | | Down |
|  | Heptanal | 0.65 | 0.961 | | Down |
|  | Hexanal | 0.683 | 0.961 | | Down |
|  | Pentadecanal- | 0.072 | 0.602 | | Up |
|  | Pentanal | 0.664 | 0.961 | | Down |
|  | Pentanal, 2-methyl- | 0.362 | 0.796 | | Down |
|  | Propanal, 2,2-dimethyl- | 0.217 | 0.707 | | Up |
|  | Tetradecanal | 0.2 | 0.707 | | Up |
|  | Undecanal | 0.184 | 0.707 | | Up |
| **Alkanes** | |  |  | |  |
|  | Decane | 1 | 1 | | Up |
|  | Dodecane | 0.064 | 0.59 | | Down |
|  | Heptane, 2,2,4,6,6-pentamethyl- | 0.467 | 0.89 | | Down |
|  | Hexane, 3,3-dimethyl- peak 2 | 0.413 | 0.84 | | Down |
|  | Octane | 0.683 | 0.961 | | Down |
|  | Oxirane, decyl- | 0.107 | 0.704 | | Up |
|  | Oxirane, methoxymethyl- | 0.155 | 0.705 | | Up |
|  | Tetradecane | 0.555 | 0.944 | | Down |
|  | Cyclohexane, 1-ethenyl-1-methyl-2,4-bis1-methylethenyl-, 1S-1-alpha,2-beta,4-beta- | 0.235 | 0.707 | | Up |
|  | Cyclopentane, 1-methyl-2-2-propenyl-, trans- | 0.927 | 0.986 | | Down |
| **Benzenoids** | |  |  | |  |
|  | Anethole | 0.927 | 0.986 | | Down |
|  | Dibutyl phthalate | 0.094 | 0.7 | | Down |
| **Carboxylic acid** | |  |  | |  |
|  | Cyclohexanecarboxylic acid | 0.235 | 0.707 | | Down |
| **Cycloalkene** | |  |  | |  |
|  | Cyclopentene | 0.361 | 0.796 | | Up |
| **Esters** | |  |  | |  |
|  | Acetate, pentyl ester | 0.2 | 0.707 | | Down |
|  | 1,2,4-Benzenetricarboxylic acid, 1,2-dimethyl ester | 0.155 | 0.705 | | Down |
|  | 1,2-Benzenedicarboxylic acid, bis2-methylpropyl ester | 0.157 | 0.705 | | Down |
|  | Adipic acid, isobutyl pent-4-en-2-yl ester | 0.518 | 0.944 | | Down |
|  | BenzenePropionate, ethyl ester | 0.495 | 0.92 | | Up |
|  | BenzenePropionate, methyl ester | 0.8 | 0.979 | | Up |
|  | Butyrate, 2-methyl-, ethyl ester | 0.927 | 0.986 | | Up |
|  | Butyrate, 2-methyl-, hexyl ester | 0.751 | 0.961 | | Down |
|  | Butyrate, 2-methyl-, methyl ester | 0.751 | 0.961 | | Up |
|  | Butyrate, 2-methyl-, propyl ester | 0.785 | 0.973 | | Up |
|  | Butyrate, 3-methyl-, hexyl ester | 0.785 | 0.973 | | Down |
|  | Butyrate, 3-methyl-, propyl ester | 0.927 | 0.986 | | Up |
|  | Butyrate, butyl ester | 0.155 | 0.705 | | Down |
|  | Butyrate, ethyl ester | 0.273 | 0.735 | | Down |
|  | Butyrate, methyl ester | 0.495 | 0.92 | | Down |
|  | Butyrate, propyl ester | 0.387 | 0.815 | | Down |
|  | Butyrate, 2-methyl-, butyl ester | 0.387 | 0.815 | | Up |
|  | Carbonic acid, 2-ethylhexyl octyl ester | 0.683 | 0.961 | | Down |
|  | Cyclohexanecarboxylic acid, butyl ester | 0.467 | 0.89 | | Up |
|  | Cyclohexanecarboxylic acid, ethyl ester | 0.217 | 0.707 | | Up |
|  | Cyclohexanecarboxylic acid, methyl ester | 0.169 | 0.707 | | Down |
|  | Cyclohexanecarboxylic acid, propyl ester | 0.678 | 0.961 | | Up |
|  | Decanoate, ethyl ester | 0.142 | 0.705 | | Down |
|  | Decanoate, methyl ester | 0.022 | 0.579 | | Down |
|  | Dodecanoate, ethyl ester | 0.014 | 0.579 | | Down |
|  | Dodecanoate, methyl ester | 0.036 | 0.579 | | Down |
|  | d-Proline, N-methoxycarbonyl-, octyl ester | 0.294 | 0.738 | | Up |
|  | Ethyl Acetate | 0.13 | 0.705 | | Down |
|  | Heptanoate, butyl ester | 0.447 | 0.89 | | Down |
|  | Heptanoate, ethyl ester | 0.24 | 0.715 | | Down |
|  | Heptanoate, methyl ester | 0.495 | 0.92 | | Down |
|  | Heptanoate, propyl ester | 0.338 | 0.784 | | Down |
|  | Hexanoate, 2-methylpropyl ester | 0.751 | 0.961 | | Down |
|  | Hexanoate, butyl ester | 0.926 | 0.986 | | Down |
|  | Hexanoate, ethyl ester | 0.525 | 0.944 | | Up |
|  | Hexanoate, hexyl ester | 0.716 | 0.961 | | Down |
|  | Hexanoate, methyl ester | 1 | 1 | | Down |
|  | Hexanoate, pentyl ester | 0.82 | 0.979 | | Down |
|  | Hexanoate, propyl ester | 0.856 | 0.979 | | Down |
|  | Hydrazinecarboxylic acid, phenylmethyl ester | 0.683 | 0.961 | | Down |
|  | Isopentyl hexanoate | 0.751 | 0.961 | | Down |
|  | Isopropyl palmitate | 0.362 | 0.796 | | Down |
|  | Methyl isoValerate | 0.856 | 0.979 | | Up |
|  | Methyl propionate | 0.142 | 0.705 | | Down |
|  | Methyl salicylate | 0.785 | 0.973 | | Down |
|  | Methyl thiolacetate | 0.362 | 0.796 | | Up |
|  | n-Caprylic acid isobutyl ester | 0.294 | 0.738 | | Down |
|  | n-Propyl acetate | 1 | 1 | | Up |
|  | Octanoate, ethyl ester | 0.155 | 0.705 | | Down |
|  | Octanoate acid, methyl ester | 0.235 | 0.707 | | Down |
|  | Valerate, ethyl ester | 0.927 | 0.986 | | Down |
|  | Valerate, pentyl ester | 0.273 | 0.735 | | Down |
|  | Valerate, propyl ester | 0.856 | 0.979 | | Up |
|  | Phthalic acid, cyclobutyl isobutyl ester | 0.107 | 0.704 | | Down |
|  | Propionate, 2-methyl-, butyl ester | 0.467 | 0.89 | | Down |
|  | Propionate, 2-methyl-, ethyl ester | 0.387 | 0.815 | | Up |
|  | Propionate, 2-methyl-, propyl ester | 0.184 | 0.707 | | Down |
|  | Propionate, butyl ester | 0.235 | 0.707 | | Down |
|  | Propionate, ethyl ester | 0.617 | 0.961 | | Up |
|  | Propionate, hexyl ester | 0.963 | 0.993 | | Down |
|  | Propionate, propyl ester | 0.555 | 0.944 | | Down |
|  | Butyrate, 3-methyl-, butyl ester | 0.273 | 0.735 | | Down |
|  | Ethyl ether | 0.927 | 0.986 | | Down |
| **Fatty acids (branched)** | |  |  | |  |
|  | Butyrate, 2-methyl- | 0.856 | 0.979 | | Down |
|  | Butyrate, 3-methyl- peak 1 | 0.253 | 0.721 | | Down |
|  | Valerate, 2-methyl- | 0.294 | 0.738 | | Down |
|  | Propionate, 2-methyl- | 0.814 | 0.979 | | Up |
| **Fatty acids (long chain)** | |  |  | |  |
|  | Stearate | 0.586 | 0.961 | | Down |
|  | Oleate | 0.467 | 0.89 | | Down |
|  | Pentadeconate | 0.891 | 0.986 | | Down |
| **Fatty acids (medium chain)** | |  |  | |  |
|  | Heptanoate | 0.963 | 0.993 | | Down |
|  | Hexanoate | 0.856 | 0.979 | | Up |
|  | Decanoate | 0.019 | 0.579 | | Down |
|  | Octanoate | 0.032 | 0.579 | | Down |
| **Fatty acids (short chain)** | |  |  | |  |
|  | Acetate | 0.294 | 0.738 | | Down |
|  | Butyrate | 0.052 | 0.579 | | Down |
|  | Valerate | 0.617 | 0.961 | | Down |
|  | Propionate | 0.392 | 0.819 | | Down |
| **Halogenated compounds** | |  |  | |  |
|  | 2,5-Cyclohexadiene-1,4-dione, 5-chloro-2,3-dimethyl-, 1-oxime, o-benzoyl- | 0.82 | 0.979 | | Down |
|  | Benzaldehyde, 2-chloro- | 0.963 | 0.993 | | Up |
| **Other Hydrocarbons** | |  |  | |  |
|  | Acetoin | 0.617 | 0.961 | | Up |
|  | Furan, 2-pentyl- | 0.079 | 0.616 | | Up |
|  | Santolina triene | 0.963 | 0.993 | | Up |
|  | Styrene | 0.555 | 0.944 | | Down |
|  | Undecane, 3-methyl- | 0.02 | 0.579 | | Down |
| **Ketones** | |  |  | |  |
|  | 2,6-Dihydroxyacetophenone, 2TMS derivative | 0.184 | 0.707 | | Up |
|  | 1-Penten-3-one, 2-methyl- | 0.75 | 0.961 | | Up |
|  | 2,3-Butanedione | 0.716 | 0.961 | | Down |
|  | 2,3-Hexanedione | 0.683 | 0.961 | | Down |
|  | 2,3-Pentanedione | 0.362 | 0.796 | | Up |
|  | 2-Butanone | 0.963 | 0.993 | | Down |
|  | 2-Butanone, 3-methyl- | 0.338 | 0.784 | | Up |
|  | 2-Decanone | 0.019 | 0.579 | | Up |
|  | 2-Heptanone | 0.683 | 0.961 | | Down |
|  | 2-Heptanone, 5-methyl- | 0.963 | 0.993 | | Up |
|  | 2-Heptanone, 6-methyl- | 0.145 | 0.705 | | Up |
|  | 2-Hexanone | 0.856 | 0.979 | | Up |
|  | 2-Nonanone or 2-Nonanone, 3-hydroxymethyl- | 0.716 | 0.961 | | Down |
|  | 2-Octanone | 0.785 | 0.973 | | Down |
|  | 2-Pentanone | 0.2 | 0.707 | | Up |
|  | 2-Pentanone, 3-methyl- | 0.555 | 0.944 | | Down |
|  | 3-Amino-2-cyclohexenone | 0.555 | 0.944 | | Down |
|  | 3-Buten-2-one, 3-methyl- | 0.004 | 0.579 | | Down |
|  | 3-Hexanone | 0.891 | 0.986 | | Down |
|  | 3-Pentanone, 2-methyl- | 0.204 | 0.707 | | Up |
|  | 5,6,7-Trimethoxy-1-indanone tentative | 0.716 | 0.961 | | Down |
|  | 5,9-Undecadien-2-one, 6,10-dimethyl- | 0.217 | 0.707 | | Up |
|  | Acetone | 0.2 | 0.707 | | Up |
|  | Acetyl valeryl | 0.617 | 0.961 | | Down |
|  | Benzyl methyl ketone | 0.273 | 0.735 | | Down |
|  | Butyrolactone | 0.235 | 0.707 | | Down |
|  | Carvone | 0.525 | 0.944 | | Down |
|  | Cyclohexanone, 5-methyl-2-1-methylethyl-, trans- | 0.439 | 0.882 | | Down |
|  | Ethanone, 1-2-furanyl- | 0.891 | 0.986 | | Up |
|  | gamma-Dodecalactone | 0.927 | 0.986 | | Down |
|  | Methyl Isobutyl Ketone | 0.927 | 0.986 | | Down |
|  | 5-Hepten-2-one, 6-methyl- | 0.65 | 0.961 | | Up |
| **Lactones** | |  |  | |  |
|  | 23H-Furanone, dihydro-5,5-dimethyl-4-3-oxobutyl- | 0.785 | 0.973 | | Down |
|  | 23H-Furanone, dihydro-5-methyl- | 0.82 | 0.979 | | Down |
|  | 23H-Furanone, dihydro-5-propyl- | 0.294 | 0.738 | | Up |
| **Nitrogen-containing compounds** | |  |  | |  |
|  | 1H-Pyrrole-2,5-dione, 3-ethyl-4-methyl- | 0.586 | 0.961 | | Down |
|  | 1H-Tetrazol-5-amine | 0.248 | 0.721 | | Up |
|  | 2-Butynedinitrile | 0.046 | 0.579 | | Up |
|  | Ethanamine, N-methyl-N-nitroso- | 0.036 | 0.579 | | Down |
|  | Formic acid hydrazide | 0.064 | 0.59 | | Down |
|  | Indole | 0.001 | 0.349 | | Up |
|  | Indole, 3-methyl- | 0.025 | 0.579 | | Up |
|  | Methylamine, N,N-dimethyl- peak 1 | 0.65 | 0.961 | | Up |
|  | Pyridine | 0.079 | 0.616 | | Up |
|  | 2-Amino-1,3-propanediol | 0.079 | 0.616 | | Down |
|  | 2-Amino-2-methyl-1,3-propanediol | 0.856 | 0.979 | | Up |
|  | 3,5-Diamino-1,2,4-triazole | 0.387 | 0.815 | | Down |
|  | 5-Acetyl-2-methylpyridine | 0.155 | 0.705 | | Up |
| **Oxygen containing compounds** | |  |  | |  |
|  | p-Cresol | 0.338 | 0.784 | | Up |
|  | Phenol | 0.82 | 0.979 | | Up |
|  | Phenol, 4,6-di1,1-dimethylethyl-2-methyl- | 0.054 | 0.58 | | Down |
|  | Phenol, 4-ethyl- | 0.927 | 0.986 | | Down |
|  | Resorcinol, TMS derivative | 0.118 | 0.705 | | Up |
| **Siloxanes** | |  |  | |  |
|  | 3,6-Dioxa-2,4,5,7-tetrasilaoctane, 2,2,4,4,5,5,7,7-octamethyl- tentative | 0.41 | 0.84 | | Up |
| **Sulphur containing compounds** | |  |  | |  |
|  | Allyl Isothiocyanate | 0.716 | 0.961 | | Down |
|  | 1-Propanol, 3-methylthio- | 0.014 | 0.579 | | Up |
|  | Carbonyl sulphide | 0.217 | 0.707 | | Down |
|  | Dimethyl sulphide | 0.155 | 0.705 | | Up |
|  | Dimethyl trisulphide | 0.041 | 0.579 | | Up |
|  | Disulphide, dimethyl | 0.032 | 0.579 | | Up |
|  | Disulphide, methyl 2-propenyl | 0.555 | 0.944 | | Up |
|  | Disulphide, methyl propyl | 0.169 | 0.707 | | Up |
|  | Ethane, 1,1-methylenebisthiobis- | 0.184 | 0.707 | | Up |
|  | Methanethiol | 0.316 | 0.773 | | Up |
|  | Methional | 0.856 | 0.979 | | Up |
|  | Pentanenitrile, 5-methylthio- | 0.716 | 0.961 | | Down |
|  | Propane, 1-isothiocyanato-3-methylthio- | 0.555 | 0.944 | | Up |
|  | Propane, 1-methylthio- | 0.294 | 0.738 | | Up |
|  | S-Methyl 3-methylbutanethioate | 1 | 1 | | Down |
|  | Sulfide, allyl methyl | 0.65 | 0.961 | | Up |
|  | Sulfurous acid, 2-ethylhexyl hexyl ester | 0.751 | 0.961 | | Down |
|  | Tetrasulfide, dimethyl | 0.118 | 0.705 | | Up |
| **Terpenoids** | |  |  | |  |
|  | 1,3,8-p-Menthatriene | 0.586 | 0.961 | | Down |
|  | 1,3-Cyclohexadiene, 5-1,5-dimethyl-4-hexenyl-2-methyl-, S-R,S- | 0.82 | 0.979 | | Down |
|  | 1,5,9-Decatriene, 2,3,5,8-tetramethyl- | 0.495 | 0.92 | | Up |
|  | 3-Carene | 0.413 | 0.84 | | Up |
|  | alpha-Cubebene | 0.064 | 0.59 | | Up |
|  | alpha-Farnesene | 0.555 | 0.944 | | Down |
|  | alpha-Phellandrene peak 1 | 0.617 | 0.961 | | Down |
|  | alpha-Pinene | 0.927 | 0.986 | | Up |
|  | alpha-Terpineol | 0.142 | 0.705 | | Up |
|  | Ar-tumerone | 0.65 | 0.961 | | Down |
|  | Benzene, 1-1,5-dimethyl-4-hexenyl-4-methyl- | 0.362 | 0.796 | | Down |
|  | Benzene, 1-1,5-dimethylhexyl-4-methyl- | 0.927 | 0.986 | | Down |
|  | beta-Bisabolene | 0.65 | 0.961 | | Up |
|  | beta-Myrcene | 0.751 | 0.961 | | Down |
|  | beta-Ocimene | 0.963 | 0.993 | | Down |
|  | beta-Pinene | 0.716 | 0.961 | | Down |
|  | beta-ylangene | 0.338 | 0.784 | | Up |
|  | Bicyclo310hex-2-ene, 2-methyl-5-1-methylethyl- | 0.65 | 0.961 | | Down |
|  | Camphene | 0.891 | 0.986 | | Up |
|  | Camphor | 0.055 | 0.58 | | Up |
|  | Caparratriene | 0.155 | 0.705 | | Down |
|  | Carene | 0.617 | 0.961 | | Down |
|  | Caryophyllene | 0.072 | 0.602 | | Up |
|  | cis-2,6-Dimethyl-2,6-octadiene | 0.338 | 0.784 | | Up |
|  | Copaene | 0.028 | 0.579 | | Up |
|  | Cubenene | 0.338 | 0.784 | | Up |
|  | D-Limonene | 1 | 1 | | Down |
|  | E-beta-Famesene | 0.683 | 0.961 | | Down |
|  | gamma-Terpinene | 0.155 | 0.705 | | Down |
|  | Humulene | 0.052 | 0.579 | | Up |
|  | Limonene | 1 | 1 | | Down |
|  | Limonene oxide, trans- | 0.273 | 0.735 | | Down |
|  | Linalool | 0.65 | 0.961 | | Down |
|  | Naphthalene, 1,2,3,4-tetrahydro-1,6-dimethyl-4-1-methylethyl-, 1S-cis- | 0.058 | 0.582 | | Up |
|  | Naphthalene, 1,2,3,5,6,8a-hexahydro-4,7-dimethyl-1-1-methylethyl-, 1S-cis- | 0.2 | 0.707 | | Up |
|  | o-Cymene | 0.387 | 0.815 | | Down |
|  | Spiro55undec-2-ene, 3,7,7-trimethyl-11-methylene-, -- | 0.891 | 0.986 | | Down |
|  | trans-alpha-Bergamotene | 0.253 | 0.721 | | Up |
|  | trans-beta-Ocimene | 1 | 1 | | Down |
|  | Cyclohexene, 1-methyl-4-1-methylethylidene- | 0.751 | 0.961 | | Down |
|  | Cyclohexene, 3-1,5-dimethyl-4-hexenyl-6-methylene-, S-R,S- | 0.316 | 0.773 | | Down |
|  | Cyclohexene, 4-ethenyl-4-methyl-3-1-methylethenyl-1-1-methylethyl-, 3R-trans- | 0.294 | 0.738 | | Up |
|  | E-1-Methyl-4-6-methylhept-5-en-2-ylidenecyclohex-1-ene | 0.65 | 0.961 | | Down |
| **Unknown** | |  |  | |  |
|  | 1,3,7-Octatriene, 2,7-dimethyl- | 0.785 | 0.973 | | Down |
|  | 1-Pentadecyne | 0.184 | 0.707 | | Up |
|  | 2,2-Bi-1,3-dioxolane peak 1 | 0.107 | 0.704 | | Down |
|  | Benzoic acid, 2-hydroxy-5-1-methyl-1-phenylethyl- | 0.184 | 0.707 | | Down |
|  | Bicyclo310hexan-3-ol, 4-methylene-1-1-methylethyl-, acetate | 0.107 | 0.704 | | Up |
|  | Bicyclo720undec-4-ene, 4,11,11-trimethyl-8-methylene- | 0.028 | 0.579 | | Up |
|  | 2,6-Dimethyl-2-trans-6-octadiene | 0.235 | 0.707 | | Up |
|  | 2,6-Dioxa-tricyclo33203,7dec-9-ene | 0.362 | 0.796 | | Up |
|  | 2E,4E-3,7-Dimethylocta-2,4-diene | 0.387 | 0.815 | | Down |
|  | 2-Hexadecene, 3,7,11,15-tetramethyl-, R-R,R-E- | 0.683 | 0.961 | | Down |
|  | 2,2,4-Trimethyl-1,3-pentanediol diisobutyrate | 0.986 | 1 | | Down |
|  | 2-Octene, 2-methyl-6-methylene- | 0.253 | 0.721 | | Up |

*P*-value calculated by t-test. *q*-value calculated by FDR using the Benjamini-Hochberg correction (adjusted *P* value cut-off 0.25).
